# Study of the Wear Resistance of Conductive Poly Lactic Acid Monofilament 3D Printed onto Polyethylene Terephthalate Woven Materials

**DOI:** 10.3390/ma13102334

**Published:** 2020-05-19

**Authors:** Prisca Aude Eutionnat-Diffo, Yan Chen, Jinping Guan, Aurelie Cayla, Christine Campagne, Vincent Nierstrasz

**Affiliations:** 1 Textile Materials Technology, Department of Textile Technology, Faculty of Textiles, Engineering and Business, University of Borås, SE- 50190 Borås, Sweden; vincent.nierstrasz@hb.se; 2 ENSAIT, GEMTEX–Laboratoire de Génie et Matériaux Textiles, F-59000 Lille, France; aurelie.cayla@ensait.fr (A.C.); christine.campagne@ensait.fr (C.C.); 3 College of Textile and Clothing Engineering, Soochow University, Suzhou 215006, China; yanchen@suda.edu.cn (Y.C.); guanjinping@suda.edu.cn (J.G.)

**Keywords:** Fused Deposition Modeling (FDM), 3D printing, Poly Lactic Acid (PLA) monofilament, polyethylene terephthalate woven fabric (PET), abrasion and electrical conductivity

## Abstract

Wear resistance of conductive Poly Lactic Acid monofilament 3D printed onto textiles, through Fused Deposition Modeling (FDM) process and their electrical conductivity after abrasion are important to consider in the development of smart textiles with preserved mechanical and electrical properties. The study aims at investigating the weight loss after abrasion and end point of such materials, understanding the influence of the textile properties and 3D printing process parameters and studying the impact of the abrasion process on the electrical conductivity property of the 3D printed conductive polymers onto textiles. The effects of the 3D printing process and the printing parameters on the structural properties of textiles, such as the thickness of the conductive Poly Lactic Acid (PLA) 3D printed onto polyethylene terephthalate (PET) textile and the average pore sizes of its surface are also investigated. Findings demonstrate that the textile properties, such as the pattern and the process settings, for instance, the printing bed temperature, impact significantly the abrasion resistance of 3D printed conductive Poly Lactic Acid (PLA) onto PET woven textiles. Due to the higher capacity of the surface structure and stronger fiber-to-fiber cohesion, the 3D printed conductive polymer deposited onto textiles through Fused Deposition Modeling process have a higher abrasion resistance and lower weight loss after abrasion compared to the original fabrics. After printing the mean pore size, localized at the surface of the 3D-printed PLA onto PET textiles, is five to eight times smaller than the one of the pores localized at the surface of the PET fabrics prior to 3D printing. Finally, the abrasion process did considerably impact the electrical conductivity of 3D printed conductive PLA onto PET fabric.

## 1. Introduction

In order to be suitable for smart textiles applications, conductive thermoplastic-based nanocomposites materials polymers deposited onto textiles through Fused Deposition Modeling (FDM) process, which can exhibit ease of processing, low cost and versatility, together with acceptable adhesion [1,2,3,4] and tensile [5] properties, have to demonstrate great wear resistances.

Many researchers have studied the wear resistance of thermoplastic-based nanocomposites materials [6,7,8] and textiles [9,10,11,12,13]. Bhimaraj et al. studied the wear resistance and friction of poly(ethylene) terephthalate filled with up to 10 wt% alumina nanoparticles and demonstrated that the addition of nanofillers increased the wear by two times and decreased the friction coefficient compared to the unfilled polymer [7]. In addition, the tribological properties of the nanocomposite materials were found to be linked to their crystallinity of the polymer and nanofiller size [8]. In their review, G. Malucelli et al. have shown that the wear performance of polymeric materials depends on several factors such as the bulk and surface properties of the polymer 3D- materials, the filler size (17, 38 and 45 nm) and shape, the homogeneity of dispersion of the filler within the matrix (polymer) and the interface filler/matrix. They found that the higher the alumina nanofiller size, the better the wear resistance. In polymer science, two mechanisms of wear, called interfacial and cohesive wear processes, may occur. Cohesive wear process includes fretting, fatigue and abrasion wears and their two key parameters are the contact stresses and contact geometry. Abrasion, which is the most common type of wear, depends on many factors such as the hardness of the materials, the applied load and the characteristics of the abrasive particles. Fatigue wear occurs from repeated stress and un-stress of the material leading to fracture (cracks) from accumulation of irreversible changes and delamination in case of growth of cracks. Fretting wear can be caused by oscillatory motions of minor amplitudes that produce small fragments on the surface leading to losses of materials. The main interfacial wears is named as transfer wear and occurs when a transfer of material (solid or liquid) happens between the two interfaces involved. Several parameters can impact it, for instance, the cohesion/adhesion between the two layers, their surface roughness and the polymer structure [6].

Furthermore, in order to have a complete understanding of the abrasion resistance of conductive thermoplastic-based nanocomposites materials deposited onto textiles through FDM process, it is also important to consider the wear resistance of textile structures. To define the abrasion resistance of textiles, different test methods are used, however, the most common are the one using the Martindale and Taber equipment (mainly used in carpet industry) [14,15]. Abrasion of textiles defines the mechanical deterioration of textile surfaces by mean of rubbing them again a rougher surface. The rubbing process is done, first, on the surface of the textile and then, its internal structure [16]. The abrasion resistance of fabrics is affected by various factors such as yarn and fiber counts and textile structure [16]. For instance, satin weave will abrade more than a twill weave due to longer floats in the weave structure and thus, more exposed yarns to abrasion which can be broken more easily [17]. In addition, the cohesion of the yarns within the structure [18,19,20,21] and the crimp of the yarn in the fabric may affect the abrasion property of the material [14,16,17,18]. In addition, various researchers have investigated the effect of the yarn twist, raw materials, yarn production and finishing process on the abrasion resistance of woven fabric [16,22,23,24]. The abrasion resistance of functional textiles through digital inkjet printing was already investigated by J. Yu et al. (2018) [25]. They found that the water contact angle of 140 °C of waterborne, fluorocarbon-free ink containing polysiloxane in the form of micro-emulsion inkjet-printed onto fabrics was maintained after 20,000 rubbing cycles.

Heretofore, no studies have been carried out on the abrasion resistance of conductive thermoplastic-based nanocomposites materials deposited onto textiles through the FDM process and also its impact on the electrical conductivity. Hence, this study aims at delivering scientific knowledge on the influence of 3D printing process parameters and the textile properties on the abrasion resistance of the conductive PLA nanocomposites materials deposited onto polyethylene terephthalate woven textiles through the FDM process and its influence on the electrical conductivity of such materials. The abrasion test was carried out following the ASTM D4966-12 (2016) [14] and modified version of ASTM D257-99 (2005) [26] standards. Finally, the understanding and interpretation of the abrasion findings was fully supported by an analysis of the mean pores size localized at the surface of the 3D-printed PLA onto PET textiles after 3D printing and one of the surface of the PET fabrics prior to 3D printing.

In this study, the designation “3D-PPOT conductive materials” is used to define 3D printed conductive polymers on textiles materials.

## 2. Materials and Methods

### 2.1. Materials

The woven fabrics used were made of polyethylene terephthalate (PET) twisted multi-filaments of Nm 40 as warp yarns and polyester monofilament of 0.2 mm in diameter as weft yarns. Nm refers to the Number of hanks of 1000 m/kg. It is utilized to know the length of a yarn per unit weight. A former study showed that these fabrics demonstrate acceptable results in terms of adhesion [1].

Conductive PLA monofilaments (Ø  =  1.75  ± 0.05 mm) were manufactured in-house by extrusion process executed in a room with a controlled temperature of 20 °C ± 0.2 and humidity of 65% ± 5. First, 2.5 wt.% of carbon black (CB) fillers were physically mixed with virgin PLA granulates and then dried in an oven at 60 °C during twelve hours. Finally, the dispersion of the CB nanoparticles in the PLA matrix was executed using a Thermo Haake rotating and intermeshing twin-screw extruder running at 100 rpm and five heating zones of 170, 175, 180, 185 and 190 °C, respectively. The monofilaments were cooled by air-pressure.

### 2.2. 3D Printing Process

**Figure 1:**
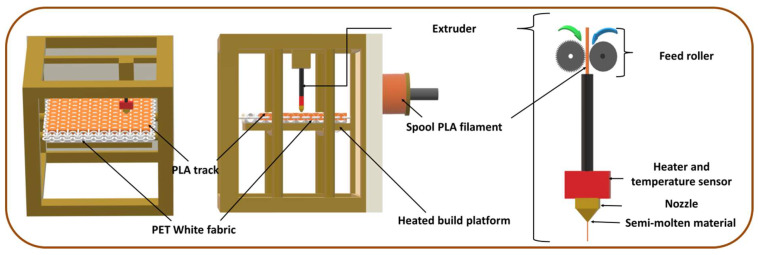
Fused deposition modeling process onto textiles.

The 3D printing manufacturing process, presented in Figure 1, was done in climatized conditions (20 °C ± 0.2 and 65% ± 5%). PET woven samples of rectangular shape (80 mm × 225 mm) were placed directly in the middle of a metallic build platform of the 3D printer (WANHAO Duplicator 4/4x, Creative Tools AB, Halmstad, Sweden) prior to the printing process. Then, a thin and rectangular layer (50 mm × 200 mm × 0.1 mm) made of conductive PLA, 1.75 mm monofilament, designed first on Rhinoceros CAD software and then imported into Simplify 3D software, was printed on each different set of woven material. The printing parameters are presented in Table 1. The distance between the head of the extruder and the surface of the textile was set during the calibration and remained constant and only the right extruder was used for all the different trials.

### 2.3. Abrasion Resistance

The abrasion resistance of the 3D-PPOT materials is tested according to ASTM D4966-12 (2016) [14]. The abrasive fabrics used for the experiments were a felt of 750 ± 50 g/m^2^ mass and 3 ± 0.3 mm thick, a polyurethane foam backing with a thickness of 3 ± 0.01 mm and a density of 29 to 31 kg/m^3^ and a standard wool abrasion fabric of 215 ± 10 g/m^2^. The weight loss percentage is determined using the Equation (1) describing the difference (in percentage) between the initial weight and the weight after 1000, 2000, 5000, 10,000, 20,000 and 30,000 cycles for the 3D-PPOT materials samples and 200, 500, 1000 and 2000 cycles for the textiles prior to 3D printing process. The end point, which is the maximum number of cycles, was also determined. Three replicates for each sample were necessary in order to guarantee an acceptable standard deviation below 10%.

(1) $$Weight\;loss\;\left(\%\right)=\frac{W_{0}-W_{X}}{W_{0}}\times 100$$

where $W_{0}$ is the initial weight of the 3D-PPOT materials or fabric, $W_{X}$ the weight of the 3D-PPOT materials or neat fabric after X number of cycles.

### 2.4. Electrical Conductivity of 3D-PPOT Material before and after Abrasion

**Figure 2:**
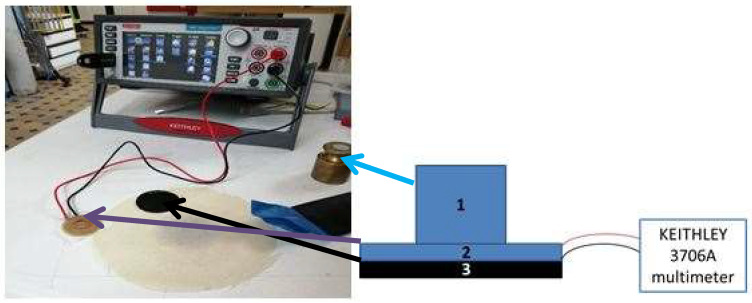
Electrical conductivity measurement system used for 3D printed conductive polymers on textiles (3D-PPOT) materials before and after abrasion (“Martindale”). (1), (2) and (3) refer to the weight of 300 grams, the electrodes and the textile material, respectively.

**Figure 3:**
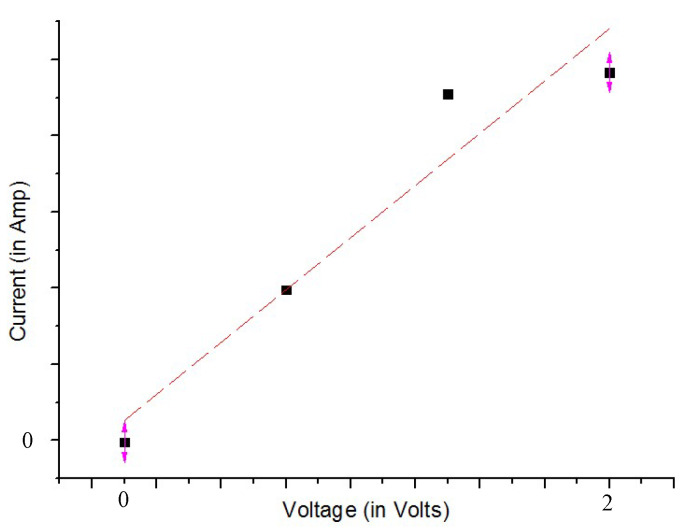
Current–voltage characteristic and their linear fitting between 0 V and 2 V.

The existing procedures used [27,28] to measure in-plane the electrical resistance of materials could not be applicable in the case of a 30 mm diameter samples made of 3D printed conducting polymer 3D printed only onto one side of the textile material. Therefore, the in-plane measurement of the electrical resistance of the 3D-PPOT samples before and after was carried-out using an in-house customized system, with an acceptable reliability, presented in Figure 2. The three centimeters diameter electrode was applied on non-abraded and abraded 3D-PPOT materials under a weight of 300 grams and connected to a Keithley 3706A digital multimeter to visualize the current–voltage characteristic for a voltage value from −0.5 Volts (V) to 3 V and a step of 0.5 V, as shown in Figure 3. The electrical conductivity is obtained by determining first the slope of the linear fitting of the curve (Figure 3) and then by using the Equations (2) and (3). Five replicates for each sample were necessary in order to guarantee an acceptable standard deviation below 10%. Three samples were used in this experiment.

(2) $${\;\sigma \;}_{S}=\frac{e}{L\times R}$$

(3) $$R=\frac{1}{f}$$

where R the electrical resistance (Ohm), e is the distance between the electrode (*e* = 5 mm), *L* is the apparent length (*L* = 39 mm) and f is the slope of the current/voltage linear fitting curve between 0 V and 2 V and ${\;\sigma \;}_{S}$ (S) is the surface electrical conductivity.

### 2.5. Pore Size measurement of Fabrics and 3D-PPOT Materials

The size of the pores localized at the surface of the 3D-PPOT materials before and after 3D-printing was measured using a capillary flow porometer—model PSM 165 from TOPAS GmbH. The sample is first immerged into a fluorocarbon solution of 16 dynes/cm and then a gas is pressurized through the sample to force the wetting liquid to go through the pores In principle, the higher the pore size, the lower the pressure at which the pores empty and reversely [29]. The pore size measurement was done to understand the abrasion of 3D-PPOT materials findings.

### 2.6. Thickness Measurement of Fabrics and 3D-PPOT Materials

The thickness of the fabrics and 3D-PPOT materials was measured using the thickness—micrometer KES-FB3 following the ISO 5084 (1996) standard [30]. Three replicates of this measurement were necessary to obtain an acceptable standard deviation.

### 2.7. Statistical Design of Experiments

Four distinct factors (printing bed temperature and weft density defined as continuous factors and fabric orientation and pattern as discontinuous ones) were defined and the order of the experiments were randomly created by Minitab 17 software. For each run executed with PET fabric and Poly Lactic Acid (PLA)/2.5twt.% Carbon Black (CB), three replicates were done for abrasion and electrical conductivity tests. The different factors are presented in Table 2, Table 3, Table 4 and Table 5. The statistical *p*-value is an important value that describes the significance of the defined factors’ impact on the measured responses, which are in our case the abrasion and electrical conductivity after abrasion of the 3D-PPOT materials. For a *p*-value below 0.05, the factor influences the responses and above 0.05 its influence is negligible. A level in factor analysis or a level of an independent variable, means that the variables can be split up into separate parts ( for example, −1,0,1).

## 3. Results and Discussion

### 3.1. Impacting Factors on the Abrasion Resistance of the PET Woven Fabrics Prior to FDM Process

**Figure 4:**
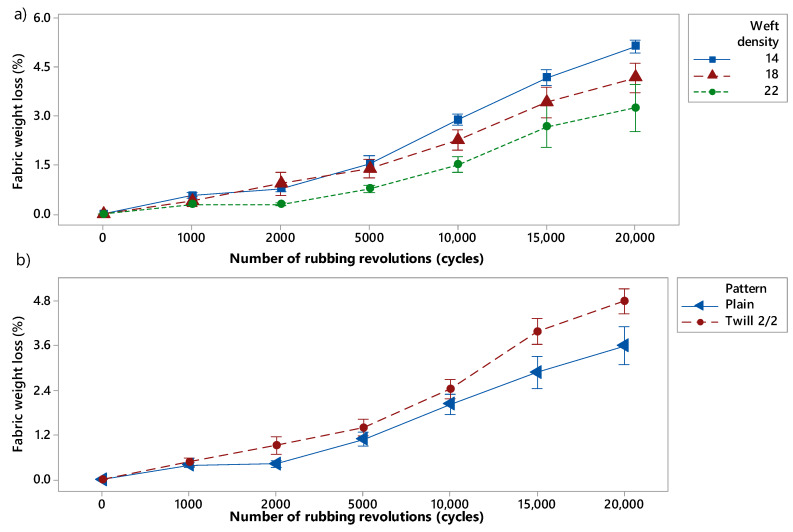
Relationship between fabric weight loss (%) prior to Fused Deposition Modeling (FDM) process and number of rubbing cycles when varying (**a**) the weft density and (**b**) the pattern. The 14, 18, 22 picks/inch are in blue, red and green, respectively) and the plain and twill patterns are in blue and red, respectively.

**Figure 5:**
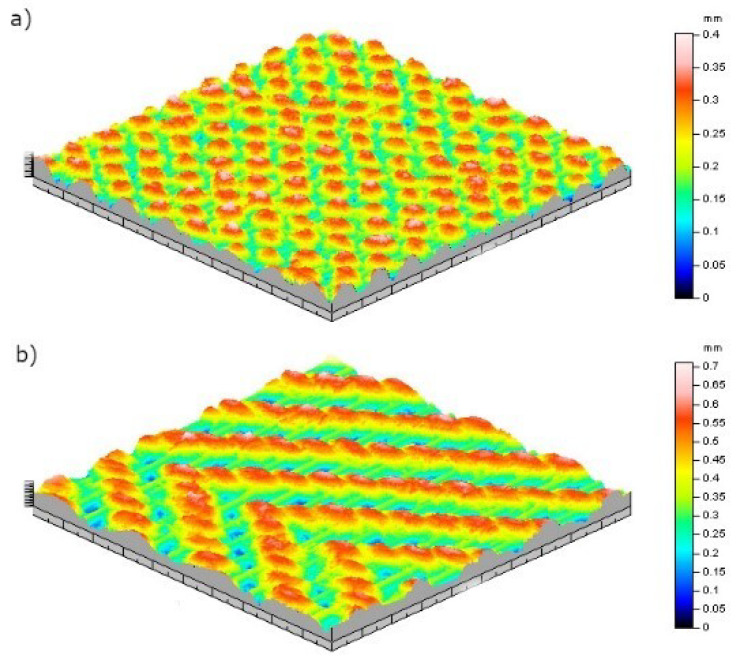
(**a**) Plain and (**b**) twill 2/2 structures visualization through profilometry method [5].

The mean values of weight loss percentage of the different samples for rubbing cycles of 1000, 2000, 5000, 10,000, 15,000 and 20,000 are given in Table A1 (Appendix A). According to the results of abrasion test, presented in Figure 4, at 2000 (*p*-value = 0.00), 5000 (*p*-value = 0.01), 10,000 (*p*-value = 0.008), 15,000 (*p*-value = 0.000) and 20,000 (*p*-value = 0.000) cycles, weave type (or pattern) had a significant effect on weight loss of woven fabrics. Furthermore, results at 2000 (*p*-value = 0.00), 5000 (*p*-value = 0.00), 10,000 (*p*-value = 0.000), 15,000 (*p*-value= 0.000) and 20,000 (*p*-value = 0.000) cycles demonstrated that the weft density significantly impact the abrasion resistance of the woven fabrics as it defined how open is the textile structure. In addition, the higher the weft density and the lower the fabric weight loss (Figure 4). Twill fabrics showed higher weight loss than plain one and the weft density presented a quadratic effect on the weight loss percentage. By observing the structure of the fabrics, it can be noticed that twill 2/2 has longer floats and less interlacing points (Figure 5). Thus, the abrasion test results indicated that textile structures which possessed lower number of interlacing and higher yarn floats presented lower abrasion resistance and higher weight loss. HK Kaynak et al. found the same trend of results in their study on the influence of fabric pattern on abrasion resistance property of woven fabrics [10].

### 3.2. Impacting Factors on the Abrasion Resistance of the 3D-PPOT Conductive Materials

**Figure 6:**
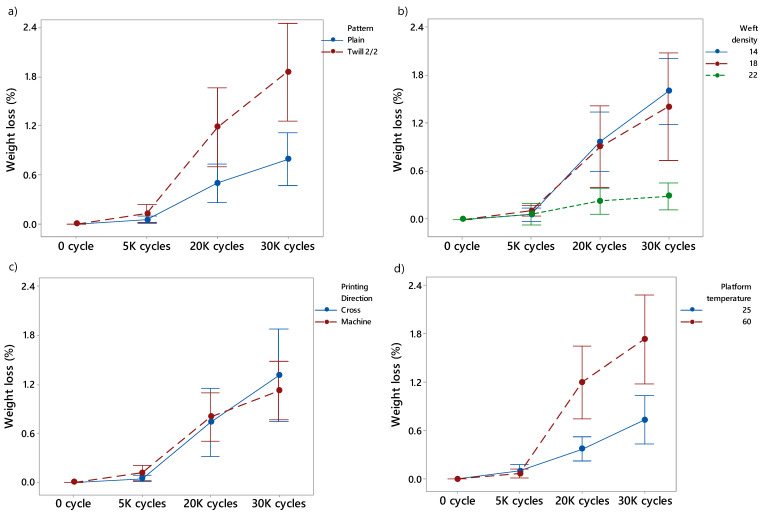
Weight loss (%) of the 3D-PPOT conductive materials after 0 cycles, 5000 (5 K), 20,000 (20 K) and 30,000 (30 K) rubbing cycles using two different patterns (**a**) plain in blue and twill 2/2 in red, three different weft densities (**b**) 14, 18 and 22 in blue, red and green, respectively, two different printing directions (**c**) cross in blue and machine in red and two different platform temperatures (**d**) 25, 60 °C in blue and red, respectively.

**Figure A1:**
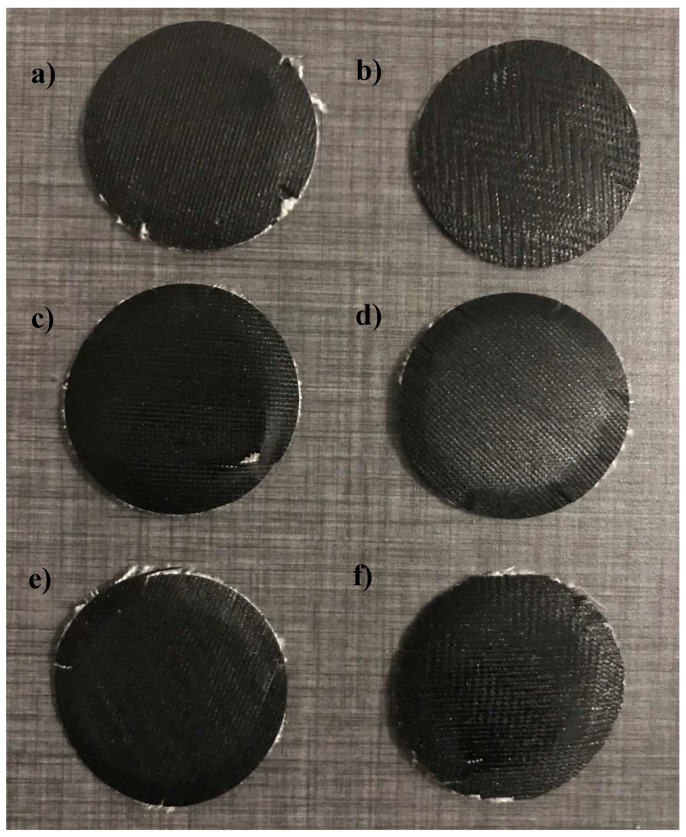
3D-PPOT samples after abrasion experiment using (**a**,**c**,**e**) 14, 18 and 22 picks/inch plain fabrics, respectively, and (**b**,**d**,**f**) 14, 18 and 22 picks/inch twill fabrics, respectively.

A visualization of the 3D-PPOT samples with different pattern and weft densities can be observed in Figure A1 (Appendix B). Visually, it is very difficult to evaluate the differences between the fabrics used, that is why the weight loss value was preferred to quantitatively analyze the influence of the FDM process. The mean values of weight loss percentage of the different samples for rubbing cycles of 5000, 20,000 and 30,000 and the ones of their end points are given in Table A2 (Appendix A). According to the weight loss percentage results of abrasion test, presented in Figure 6, the weave type or pattern (*p*-value < 0.01 above 20,000 cycles), the platform temperature (*p*-value = 0.000 above 20,000 cycles) and the weft density (*p*-value < 0.05 above 30,000 cycles) were factors which impacted the weight loss of the 3D-PPOT materials the most. In addition, the printing direction did not influence the weight loss at all. The lowest weight loss (in percentage) was obtained when using plain fabric as substrate, the lowest platform temperature (25 °C) and highest weft density (22 picks/inch). Due to its structure, plain fabric is more compact than twill 2/2 and that enhances the cohesion between the yarns and the fibers after FDM process. Thus, the 3D-PPOT materials using plain fabric as substrate is much harder to abrade and fracture compared to another one which presents floats such as twill 2/2. Similar trend can be noticed with the plain and twill 2/2 fabrics prior to 3D printing (Figure 4). In addition, in another study [1], it was previously shown that the adhesion force between the conductive PLA printed track was much higher using twill 2/2 substrate compared to plain one. It means that during FDM process, the PLA polymer may have penetrated more the twill 2/2 structure due to its higher porosity and roughness. This seemed to have a negative effect on its abrasion resistance. Similar explanation can be used to understand the better abrasion property of the 3D-PPOT materials when using denser fabric (22 picks/inch). Higher weft density demonstrated lower adhesion property while it reduced the weight loss after abrasion test due to a reduced accessibility to the whole structure of the textiles through the thickness.

**Figure 7:**
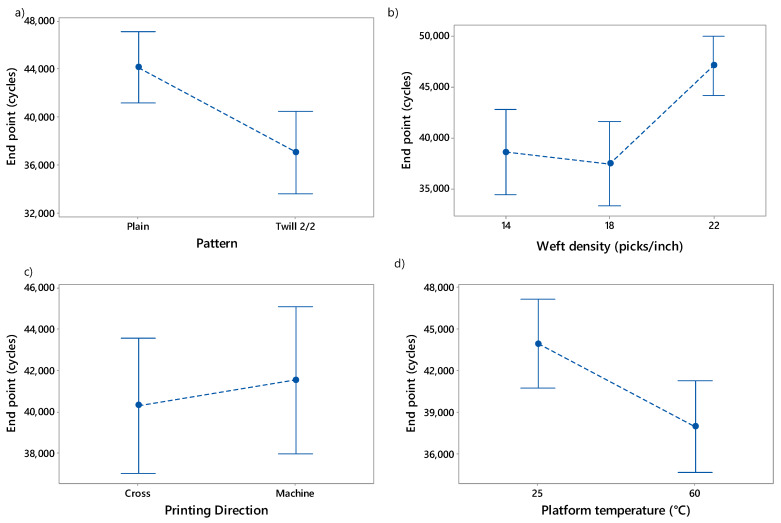
Influence of (**a**) fabric pattern, (**b**) weft density, (**c**) printing direction and (**d**) platform temperature on the end point which is the maximum number of cycles (cycles) of 3D-PPOT conductive materials.

Furthermore, the weave type or pattern (*p*-value = 0.001), the weft density (*p*-value = 0.000) and the platform temperature (*p*-value = 0.002) revealed to be the factors which influenced the end point (maximum number of cycles) of the 3D-PPOT materials the most (Figure 7). The printing direction (machine or cross) did not impact the end point at all. Similar to the weight loss analysis, higher end points were obtained using plain as a substrate, the highest weft density (the densest fabric) and the lowest platform temperature.

### 3.3. Comparison of the Abrasion Resistance of the 3D-PPOT Materials and the PET Woven Fabrics

**Figure 8:**
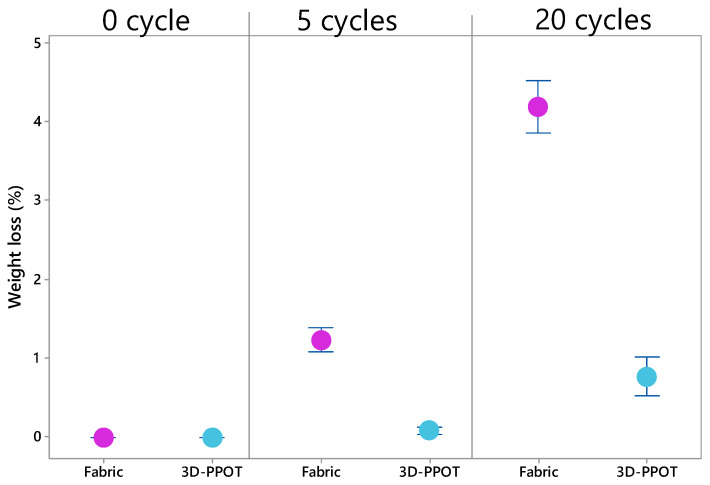
Weight loss (%) of woven fabric and 3D-PPOT material after 0, 5 and 20 cycles.

**Figure A2:**
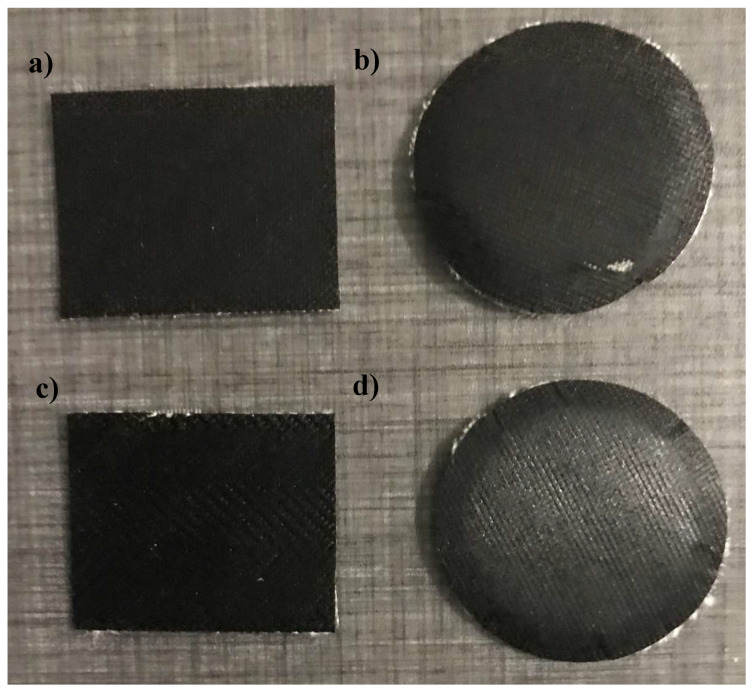
D-PPOT samples (**a**,**c**) before and (**b**,**d**) after abrasion experiment. (**a**,**b**) represent 3D-PPOT made of 18 picks/inch plain fabrics; (**c**,**d**) ones made from 18 picks/inch twill fabrics.

The weight loss and the maximum number of rubbing cycles were determined for the fabrics and the 3D-PPOT materials separately. The abrasion resistance of the 3D-PPOT materials revealed to be much better than the original woven fabrics. The weight loss decreased by about twice at 5000 rubbing cycles and by about four at 20,000 rubbing cycles (Figure 8) and there is an enhancement of the end point of the material. It means that FDM process onto textiles using conductive PLA filament improved the abrasion performance of the textiles. These findings can also be confirmed by visualizing the 3D-PPOT samples prior to and after abrasion test (Figure A2 in Appendix B). There is no significant differences between the samples. Indeed, by applying a thin layer of conductive PLA on the textile surface, the cohesion between the fibers is enhanced resulting in a stiffer and more stable surface [5] which can better resistance to abrasion.

### 3.4. Effect of 3D Printing and Textiles’ Properties on Pore Sizes of Textile Fabrics and 3D-PPOT Materials

**Figure 9:**
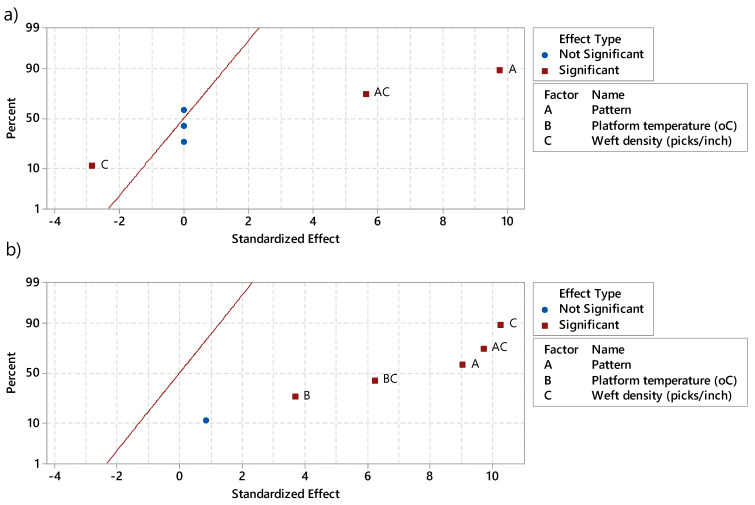
Effect of (A) pattern, (B) platform temperature in °C, and (C) weft density in picks/inch on (**a**) pore size of the fabric prior to 3D printing process and (**b**) 3D-PPOT materials after 3D printing process, obtained through pareto analysis of Minitab 17. AC and BC represent the interaction between the factors A and C and, B and C, respectively.

Prior to 3D printing process (Figure 9a), the pattern and the weft density of PET fabrics revealed to significantly impact its average pore size. This finding was already proven in the past by researchers [1]. It can be easily understood that the pattern as well as the weft density define the arrangement of the fibers in the structure. Indeed, fabrics which have a twill pattern are more open compared to those with plain ones. Furthermore, the higher the weft density, the more porous the textile structure and surface will be [1].

After 3D printing process (Figure 9b), the three factors which are pattern, platform temperature and weft density influenced meaningfully the pore size of the 3D-PPOT materials. It was already demonstrated that the platform temperature had an influence on the adhesion strength between the fabric and the printed layer due to polymer diffusion through the materials which led to stronger mechanical interlocking. If this phenomenon occurred, the pores of the structures decrease considerably as they are filled with polymeric materials. Moreover, it could be noticed that the following interactions pattern/weft density and platform temperature/weft density needed also to be considered when predicting the pore size of 3D-PPOT materials.

**Figure 10:**
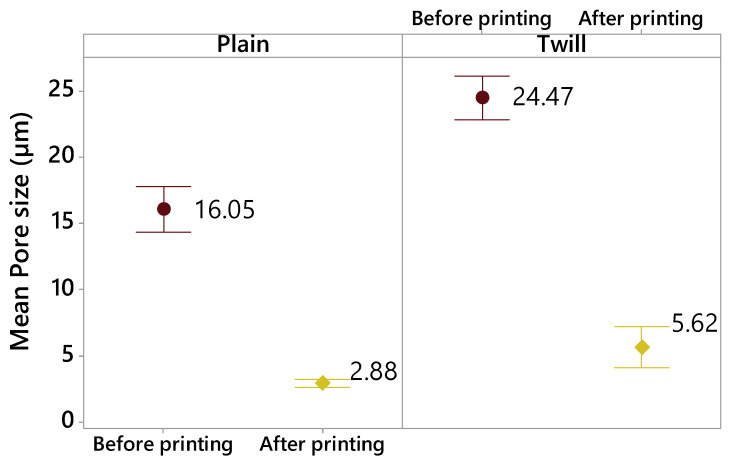
Mean pore size (µm) of the pores localized at the surface of the textile fabrics before 3D printing (in purple) and the 3D-PPOT materials after 3D printing (in yellow) depending on the fabric pattern (plain and twill).

**Figure 11:**
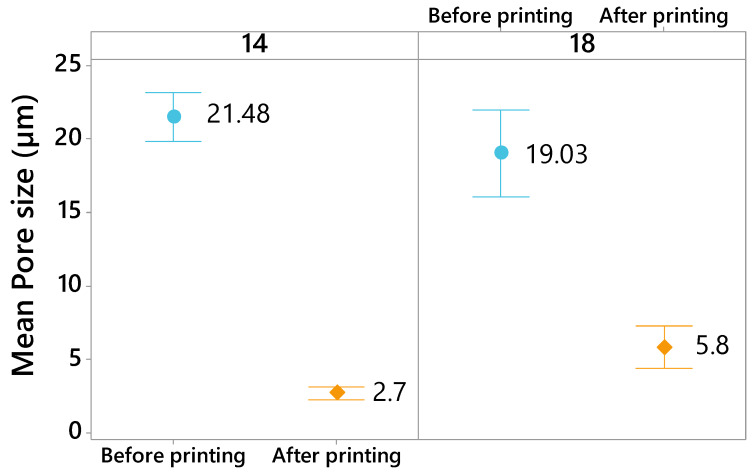
Mean pore size (µm) of the pores localized at the surface of the textile fabrics before 3D printing (in blue) and the 3D-PPOT materials after 3D printing (in orange) depending on the weft density of the fabric (14 and 18 pick/inch).

In addition, the effect of the 3D printing on the mean pore size of textiles after deposition process was investigated (Figure 10). After 3D printing, the 3D PPOT materials remain porous with smaller pores compared to the original fabrics. The deposition process onto the textiles decreased the mean pore size of the textile fabrics by about 82% (from 16.05 µm to 2.88 µm) and 77% (from 24.47 µm to 5.62 µm) for plain and twill fabrics, respectively. Indeed, this process closes the pores localized at the surface of the textile materials, which is why the mean pores size (average size of pores) decreases. The 3D-PPOT samples made of twill fabrics are more porous before and after 3D printing process. Similar observations are made for 3D-PPOT samples with different weft densities (Figure 11). Indeed, the mean pore size reduced from 21.48 µm to 2.7 µm (for 14 picks/inch) and from 19.03 µm to 5.8 µm (for 18 picks/inch).

### 3.5. Effect of 3D Printing and Textiles’ Properties on Thickness of Textile Fabrics and 3D-PPOT Materials

**Figure 12:**
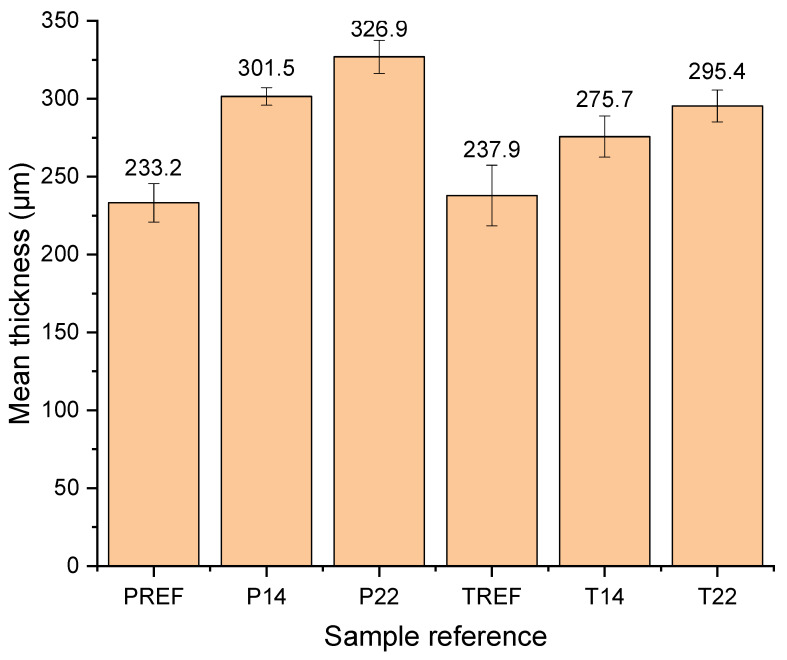
Mean thickness (µm) of the textile fabrics before 3D printing (PREF and TREF) and the 3D-PPOT materials after 3D printing (P14, P22, T14 and T22). PREF and TREF stands for plain fabric reference and twill fabric reference, respectively. P14, P22, T14 and T22 are designed as the following 3D-PPOT materials: plain—14 picks/inch, plain—22 picks/inch, twill—14 picks/inch, twill—22 picks/inch.

The mean thicknesses of the fabrics and the 3D printed conductive PLA onto textiles were measured and presented in Figure 12. Two main observations of the findings are important to highlight. First, the thickness of the material decreased from 301.5 µm to 275.7 µm in respect of 3D printing on a weave fabric with plain and twill patterns, respectively, printed and weaved in the same conditions. Additionally, it was shown that a layer of conductive PLA printed with the same conditions on twill or plain fabric with higher weft densities trend to increase the thickness of the overall materials. These results could easily be explained by the higher penetration of the polymer nanocomposites through the textiles with larger pores, which are in our case the twill fabrics. The higher the weft density of the fabrics, the bigger the pores [1]. Additionally, the findings may be explained by the compression force applied on the fabrics during 3D printing process combined with the high temperature.

**Figure 13:**
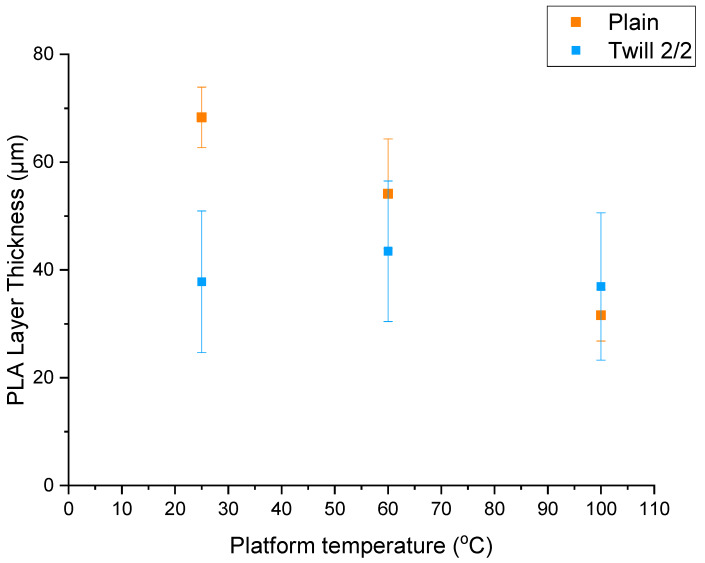
Poly lactic acid (PLA) track thickness (µm) depending on the platform temperature of the 3D printer (25, 60 and 100 °C) for plain fabrics (in orange) and twill fabric (in blue).

The effect of the platform temperature on the thickness of 3D printed conductive PLA track was also evaluated. The thickness of 3D printed PLA layer was calculated by subtracting the thickness of the original fabrics to the thickness of the 3D-PPOT materials (Figure 13). It could be noticed for both patterns (plain and twill) that an increase of the platform temperature above the glass transition of PLA (60 °C) led to decrease the thickness of the PLA layer. This finding is linked to the adhesion properties of the 3D-PPOT materials. Indeed, it was already proven that a rise of the printing bed temperature above the glass transition of the polymer enhanced the adhesion between the textile and the printed layer [1] and as a result, diminished the thickness of the 3D-PPOT materials.

### 3.6. Effect of the Abrasion on the Electrical Conductivity of the 3D-PPOT Materials

**Figure 14:**
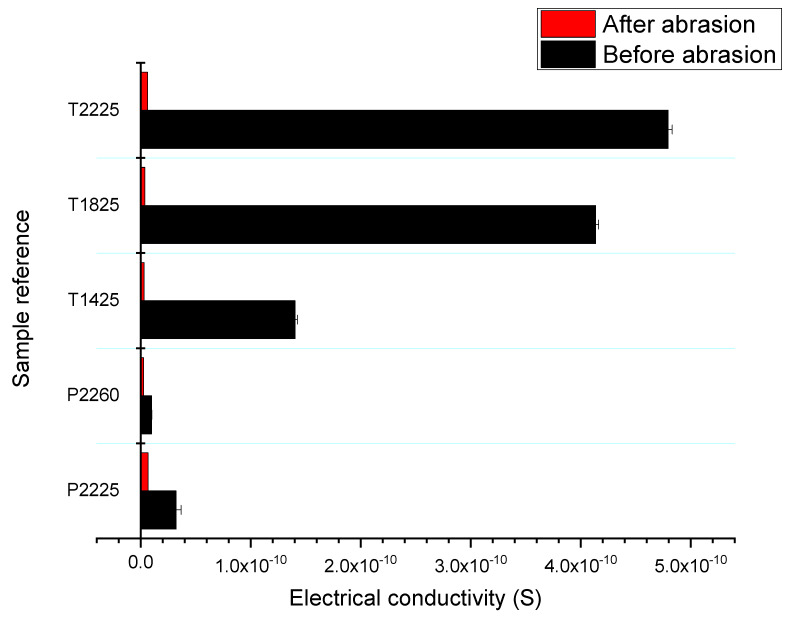
Electrical conductivity (S) before and after abrasion (20 K cycles) for the 3D-PPOT materials samples P2260, P2225, T1425, T1825 and T2225. P2260, P2225, T1425, T1825 and T2225. P2260 is designed as the following: plainof 22 picks/inch weft density and printed at a platform temperature of 60 °C.

The abrasion process affects considerably the electrical conductivity of the 3D-PPOT materials especially when using twill fabrics as textile supports (Figure 14). The higher the weft density, the better the electrical conductivity prior to abrasion. However, the gap between the values of electrical conductivity before and after abrasion is larger: Increasing the platform temperature (up to its glass transition) trends to decrease the electrical conductivity of the 3D-PPOT materials.

**Figure 15:**
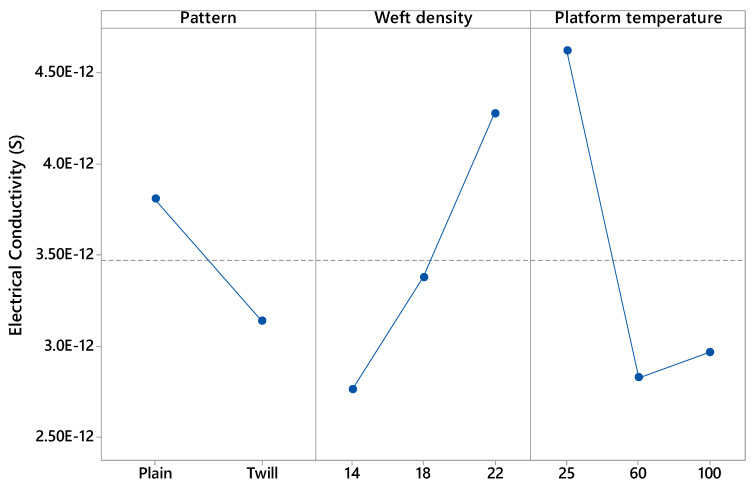
Influence of pattern (plain and twill), weft density (14,18 and 22 picks/inch) and platform temperature (25, 60 and 100 °C) on the electrical conductivity (S) of the samples after abrasion (20 K cycles). In y-axis the following scientific notation 2.50E-12 means 2.50 × 10^−12^.

In addition, the effect of the pattern, weft density and platform temperature on the electrical conductivity after 20,000 rubbing cycles was presented in Figure 15. Plain fabrics have better electrical conductivities than twill ones after abrasion. The weft density of the fabric and platform temperature presented a significant effect. The higher the weft density, the higher the electrical conductivity and the platform temperature impact quadratically the electrical conductivity after abrasion.

## 4. Conclusions

In this study, the abrasion resistance of 3D-PPOT materials made of polymeric material 3D printed onto textiles, through Fused Deposition Modeling (FDM) process was investigated. The weave type (or pattern), the weft density and the platform temperature revealed to be the factors which influenced the weight loss and the end point of the 3D-PPOT materials the most. The printing direction did not affect the abrasion resistance at all. Better abrasion resistance was obtained when using plain as a substrate, the highest weft density (most dense fabric) and the lowest platform temperature. While porous and rough textile structures used as substrate in the FDM process would improve the adhesion properties between the two layers of the 3D-PPOT materials, they would decrease their abrasion resistance. Finally, due to higher compacity of the surface structure, lower porosity and stronger fiber-to-fiber cohesion of the 3D-PPOT conductive materials produced from FDM process, they revealed to have higher abrasion resistance and lower weight loss after abrasion compared to the original unprinted fabrics. Finally, the abrasion process did considerably influence the electrical conductivity of 3D printed conductive Poly Lactic Acid onto textiles.

## Figures and Tables

**Table 1:** Printing process parameters.

Parameters	Values (unit)
Infill percentage	20 (%)
Z offset (distance between the head)	0 (mm)
Printing speed	3600 (mm·min^−1^)
Extruder (or printing) diameter	0.4 (mm)
Extruder temperature	250 (°C)

**Table 2:** Factors of the statistical design of experiments and their levels for abrasion measurements.

Factors	Name	Level		
		−1	0	1
A	Pattern	Plain	-	Twill
B	Weft density (picks/inch)	14	18	22
C	Fabric orientation	Cross	-	Machine
D	Printing bed temperature (°C)	25	-	60

**Table 3:** Factors of the statistical design of experiments and their levels for mean pore size measurement.

Factors	Name	Level	
		−1	1
A	Pattern	Plain	Twill
B	Weft density (picks/inch)	14	18

**Table 4:** Factors of the statistical design of experiments and their levels for thickness measurements.

Factors	Name	Level		
		−1	0	1
A	Pattern	Plain	-	Twill
B	Weft density (picks/inch)	14	-	22
C	Printing bed temperature (°C)	25	60	100

**Table 5:** Factors of the statistical design of experiments and their levels for electrical conductivity measurements.

Factors	Name	Level		
		−1	0	1
A	Pattern	Plain	-	Twill
B	Weft density (picks/inch)	14	18	22
C	Fabric orientation	Cross	-	Machine
D	Printing bed temperature (°C)	25	60	100

## Appendix A

**Table A1:** Mean weight loss (%) after different rubbing cycles: 1000, 2000, 5000, 10,000, 15,000 and 20,000. A, B, C and D are the pattern, the weft density, the fabric orientation and the printing bed temperature, respectively.

A	B	C	D	Mean Weight Loss (%) after Different Rubbing Cycles					
				1000	2000	5000	10,000	15,000	20,000
*−1*	*1*	*1*	*−1*	0.31	0.31	0.55	1.05	1.33	1.76
*1*	*0*	*−1*	*1*	0.56	0.87	1.41	2.91	4.04	4.98
*1*	*1*	*1*	*−1*	0.28	0.28	1.02	1.98	4.01	4.74
*−1*	*1*	*−1*	*−1*	0.31	0.31	0.55	1.05	1.33	1.76
*−1*	*0*	*−1*	*−1*	0.58	0.69	1.65	2.86	4.31	5.28
*1*	*0*	*−1*	*−1*	0.56	0.87	1.41	2.91	4.04	4.98
*1*	*−1*	*1*	*−1*	0.56	1.61	1.75	2.39	3.87	4.65
*−1*	*1*	*1*	*1*	0.31	0.31	0.55	1.05	1.33	1.76
*1*	*−1*	*−1*	*−1*	0.56	1.61	1.75	2.39	3.87	4.65
*−1*	*−1*	*1*	*−1*	0.24	0.24	1.03	2.13	2.98	3.71
*−1*	*−1*	*−1*	*−1*	0.24	0.24	1.03	2.13	2.98	3.71
*1*	*0*	*1*	*−1*	0.56	0.87	1.41	2.91	4.04	4.98
*1*	*−1*	*1*	*1*	0.56	1.61	1.75	2.39	3.87	4.65
*−1*	*0*	*1*	*1*	0.58	0.69	1.65	2.86	4.31	5.28
*1*	*1*	*−1*	*−1*	0.28	0.28	1.02	1.98	4.01	4.74
*1*	*1*	*1*	*1*	0.28	0.28	1.02	1.98	4.01	4.74
*−1*	*−1*	*1*	*1*	0.24	0.24	1.03	2.13	2.98	3.71
*−1*	*−1*	*−1*	*1*	0.24	0.24	1.03	2.13	2.98	3.71
*−1*	*0*	*−1*	*1*	0.58	0.69	1.65	2.86	4.31	5.28
*−1*	*1*	*−1*	*1*	0.31	0.31	0.55	1.05	1.33	1.76
*1*	*1*	*−1*	*1*	0.28	0.28	1.02	1.98	4.01	4.74
*1*	*−1*	*−1*	*1*	0.56	1.61	1.75	2.39	3.87	4.65
*−1*	*0*	*1*	*−1*	0.58	0.69	1.65	2.86	4.31	5.28
*1*	*0*	*1*	*1*	0.56	0.87	1.41	2.91	4.04	4.98

**Table A2:** Mean weight loss (%) after different cycles: 1000, 2000, 5000, 10,000, 15,000 and 20,000 and maximum number of cycles (end point). A, B, C and D are the pattern, the weft density, the fabric orientation and the printing bed temperature, respectively.

A	B	C	D	Mean Weight Loss (%) after Different Cycles			Mean End Point (Cycles)
				5000	20,000	30,000	-
*−1*	*1*	*1*	*−1*	0.00	0.00	0.00	50,000
*1*	*0*	*−1*	*1*	0.22	3.33	4.31	30,000
*1*	*1*	*1*	*−1*	0.44	0.44	0.59	50,000
*−1*	*1*	*−1*	*−1*	0.00	0.00	0.00	50,000
*−1*	*0*	*−1*	*−1*	0.16	0.22	0.23	50,000
*1*	*0*	*−1*	*−1*	0.00	0.93	2.25	30,000
*1*	*−1*	*1*	*−1*	0.44	0.27	1.09	50,000
*−1*	*1*	*1*	*1*	0.02	0.42	0.49	50,000
*1*	*−1*	*−1*	*−1*	0.00	1.18	2.13	30,000
*−1*	*−1*	*1*	*−1*	0.04	0.63	0.91	50,000
*−1*	*−1*	*−1*	*−1*	0.00	0.00	0.00	50,000
*1*	*0*	*1*	*−1*	0.21	0.28	0.31	50,000
*1*	*−1*	*1*	*1*	0.00	1.96	1.96	30,000
*−1*	*0*	*1*	*1*	0.23	1.39	2.11	30,000
*1*	*1*	*−1*	*−1*	0.00	0.23	0.36	40,000
*1*	*1*	*1*	*1*	0.00	0.99	1.01	50,000
*−1*	*−1*	*1*	*1*	0.07	2.44	2.97	30,000
*−1*	*−1*	*−1*	*1*	0.06	0.37	1.67	40,000
*−1*	*0*	*−1*	*1*	0.03	0.03	0.03	50,000
*−1*	*1*	*−1*	*1*	0.00	0.34	0.47	50,000
*1*	*1*	*−1*	*1*	0.24	0.70	0.98	30,000
*1*	*−1*	*−1*	*1*	0.00	1.00	2.01	30,000
*−1*	*0*	*1*	*−1*	0.02	0.12	0.62	30,000
*1*	*0*	*1*	*1*	0.00	0.96	1.39	30,000
